# Radiation dosimetry and pharmacokinetics of the tau PET tracer florzolotau (18F) in healthy Japanese subjects

**DOI:** 10.1007/s12149-023-01828-x

**Published:** 2023-03-09

**Authors:** Masaomi Miyamoto, Chio Okuyama, Shinya Kagawa, Kuninori Kusano, Masaaki Takahashi, Keisuke Takahata, Ming-Kuei Jang, Hiroshi Yamauchi

**Affiliations:** 1 APRINOIA Therapeutics Inc., Tokyo, Japan; 2 Shiga Medical Center Research Institute, Moriyama, Japan; 3 Institute for Quantum Medical Science, Quantum Life and Medical Science Directorate, National Institutes for Quantum Science and Technology, Chiba, Japan; 4 Department of Psychiatry, Graduate School of Medicine, Kyoto University, Kyoto, Japan

**Keywords:** Florzolotau (18F) (APN-1607, PM-PBB3), Tau PET ligand, Dosimetry study, Pharmacokinetics, Effective dose

## Abstract

**Objective:**

Abnormal aggregation of tau in the brain is a major contributing factor in various neurodegenerative diseases. Florzolotau (18F) (florzolotau, APN-1607, PM-PBB3) has been shown to be a probe for tau fibrils in an animal model and patients with Alzheimer’s disease and those with non-Alzheimer’s disease tauopathies. The objective of this study is to evaluate the safety, pharmacokinetics, and radiation dose following a single intravenous administration of florzolotau in healthy Japanese subjects.

**Methods:**

Three healthy male Japanese subjects aged between 20 and 64 were enrolled in this study. Subjects were determined to be eligible based on the screening assessments at the study site. Subjects received a single intravenous dose of 195.0 ± 0.5 MBq of florzolotau and underwent the whole-body PET scan 10 times in total to calculate absorbed doses to major organs/tissues and effective dose. Radioactivities in whole blood and urine were also measured for pharmacokinetic evaluation. Absorbed doses to major organs/tissues and effective dose were estimated using the medical internal radiation dose (MIRD) method. Vital signs, electrocardiography (ECG), and blood tests were done for safety evaluation.

**Results:**

The intravenous injection of florzolotau was well tolerated. There were no adverse events or clinically detectable pharmacologic effects related to the tracer in any subjects. No significant changes in vital signs and ECG were observed. The highest mean initial uptake at 15 min after injection was in the liver (29.0 ± 4.0%ID), intestine (4.69 ± 1.65%ID), and brain (2.13 ± 0.18%ID). The highest absorbed dose was 508 μGy/MBq of the gallbladder wall, followed by the liver of 79.4 μGy/MBq, the pancreas of 42.5 μGy/MBq, and the upper large intestine of 34.2 μGy/MBq. The effective dose was calculated as 19.7 μSv/MBq according to the tissue weighting factor reported by ICRP-103.

**Conclusion:**

Florzolotau intravenous injection was well tolerated in healthy male Japanese subjects. The effective dose was determined as 3.61 mSv when 185 MBq florzolotau was given.

## Introduction

According to the Alzheimer’s Disease International, the number of people with dementia in the world is estimated to be around 50 million in 2020 and is expected to nearly triple to 152 million by 2050 [1]. The number of Alzheimer’s disease accounts for about two-thirds of all dementia cases. The neuropathological hallmarks of Alzheimer’s disease include senile plaque consisting of amyloid-beta (Aβ) peptides, neurofibrillary tangles consisting of tau fibrils, and brain atrophy. Among biomarkers for Alzheimer’s disease, positron emission tomography (PET) is a useful tool in that it is non-invasive and able to quantitatively evaluate the uptake of the ligand by brain regions as well as to evaluate the brain distribution of the target molecule. Aβ aggregates have been successfully imaged in Alzheimer’s disease patients using high-affinity ^11^C- and ^18^F-labeled PET tracers (PIB and florbetapir) [2, 3]. The ability to image brain amyloid offered a significant advance for the diagnosis of neurodegenerative conditions. Currently, radiopharmaceuticals for Aβ PET imaging including florbetapir (18F), flutemetamol (18F), and florbetaben (18F) are commercially available [3–5]. However, based on the analyses of postmortem brains or results of Aβ PET imaging, it is known that Aβ begins to accumulate as early as 15 years before the onset of symptoms, then reaches a plateau at the stage of mild cognitive impairment (MCI), and that Aβ is found with advancing age even in elderly persons without cognitive impairment. Thus, Aβ does not necessarily reflect the symptomatic progress of Alzheimer’s disease. Therefore, it is not necessarily useful biomarker for diagnosis or determination of the therapeutic treatment effect [6].

Meanwhile, previous research has shown that intracellular and extracellular accumulation of tau protein is related to neurodegeneration or clinical symptoms of Alzheimer’s disease [7, 8]. Tau is a microtubule-associated protein mainly expressing in neurocytes. There are 6 isoforms of tau resulting from alternative splicing and they are broadly categorized into tau proteins with three microtubule-binding sites (3-repeat tau) and tau proteins with four microtubule-binding sites (4-repeat tau). In addition to Alzheimer’s disease, accumulation of tau protein is found in the brains of patients, such as Pick’s disease, progressive supranuclear palsy, corticobasal degeneration, and so on, where Aβ pathologies are not observed. Types of tau isoforms accumulating in the brain vary depending on disease type, both 3-repeat and 4-repeat tau accumulate in Alzheimer’s disease, 3-repeat tau mainly accumulates in Pick’s disease, and 4-repeat tau mainly accumulates in progressive supra-nuclear palsy and cortico-basal degeneration [9, 10]. Tau PET imaging of the living brain is expected to be used not only for diagnosis of these diseases named as tauopathies but also for subject screening in clinical studies of therapeutic drugs or for evaluation of treatment effects of new anti-dementia drugs that prevent neuronal cell death by inhibiting tau accumulation. Flortaucipir, the only FDA-approved tau PET tracer, is known to bind to monoamine oxidase B (MAO-B) and/or monoamine oxidase A (MAO-A) [11, 12]. These off-targets prevent the tracer from being used widely. Thus, multiple investigational tau PET ligands including florzolotau (18F) (APN-1607, PM-PBB3) have been currently under development as the second-generation tau PET tracers.

Florzolotau is a new chemical entity discovered at Institutes for Quantum Life and Medical Science at National Institutes for Quantum Science and Technology, Chiba, Japan. Results from nonclinical and clinical studies so far have shown that florzolotau binds to tau lesions with high affinity and less non-specific off-target binding including MAO-B and MAO-A, and shown the probe captures not only Alzheimer’s disease but also non-Alzheimer-type tau pathologies with a dynamic range sufficient for differentiation [13, 14], and these findings have been confirmed by other investigators [15]. The binding sites of florzolotau in the β-helix of paired helical and straight filaments of tau from Alzheimer’s disease have been reported [16]. Other investigational products, such as MK-6240 and PI-2620, can visualize tau lesions of Alzheimer’s disease and other diseases where 3-repeat and 4-repeat tau are mixing; however, they are scarcely able to detect tau lesions of tauopathies where only 4-repeat tau accumulates, such as progressive supra-nuclear palsy and cortico-basal degeneration [17, 18].

The dosimetry study on florzolotau was conducted in the US and Taiwan [19, 20]. Although the safety and the dosimetry data on florzolotau were used for Investigational New Drug (IND) application submitted to regulatory agencies, both the study results were not published. The objective of this Phase 1 study of florzolotau (APN-1607-J01) is to evaluate the safety, pharmacokinetics, absorbed doses to major organs/tissues and effective dose of single intravenous administration of florzolotau in healthy adult male Japanese subjects, and publish the study results.

## Methods and materials

### Determination of sample size

The sample size of this study was not based on a statistical power calculation. This study was intended to assess the safety and pharmacokinetics of florzolotau in healthy male subjects and to estimate absorbed doses and effective doses. The absorbed doses and the effective doses of florzolotau in healthy subjects had been evaluated in the previous phase 1 study in the US (Study 4216, *n* = 2, [19]) and the clinical research in Taiwan (Study 16083111, *n* = 12, [20]). The number of subjects in this study was determined to be 3, a minimum number to compare the absorbed doses and effective doses between different ethnic populations.

### Subjects

This study was conducted at the Shiga Medical Center Research Institute in Moriyama, Japan. The clinical study protocol including amendments, informed consent form (ICF), and all other study-related documents were reviewed and approved by an Institutional Review Board of Shiga General Hospital, and the study was conducted in compliance with the study protocol, Good Clinical Practice (GCP) as set forth in the International Conference on Harmonization (ICH) guidelines on GCP (ICH E6(R2)) and registered with the Japan Pharmaceutical Information Center (JapicCTI-205302). The subject inclusion criteria included being male Japanese, age between 20 and 64, the ability to provide his written informed consent, and not diagnosed with any disease affecting participation in the study and being normal at the screening tests of vital signs, electrocardiogram, hematology, and blood biochemical tests. Four subjects were screened, and 3 subjects with height of 175.8 ± 8.7 cm and body weight of 75.7 ± 8.7 kg were enrolled. The fourth subject was enrolled as a spare subject and did not undergo the studies according to the prior arrangement in the study protocol.

### Preparation of florzolotau

Florzolotau was produced by Ibaraki laboratory of Fujifilm Toyama Chemical Company (currently PDRadiopharma Inc., Tokyo, Japan). Florzolotau was synthesized by the NEPTIS perform synthesizer (Ora, Neuville, Belgium) by the reaction of tosylate precursor of florzolotau, (3-((2-((1*E*,3*E*)-4-(6-(methylamino)pyridin-3-yl)buta-1,3-dien-1-yl)benzo[*d*]thiazol-6-yl)oxy)-2-((tetrahydro-2H-pyran-2-yl)oxy)propyl4-methylbenzenesulfonate (APRINOIA Therapeutics Inc., Tokyo, Japan). The cyclotron program sequence was initiated, bombarding the ^18^O target water according to a preset beam current and time, and producing ^18^F-fluoride ions by the ^18^O(p,n)^18^F nuclear reaction. After the bombardment, the target water^18^F-fluoride ion was transferred to the NEPTIS synthesizer. ^18^F-fluoride ion dissolved in^18^O enriched target water was stored in a conical tube (^18^F tube) and then passed through a QMA ion exchange column [pre-activated with water for injection (5 mL), K_2_CO_3_ solution (5 mL), and water for injection (5 mL)]. The ^18^F-fluoride ion in the target water was trapped onto the ion exchange column. The ^18^F-fluoride ion was eluted from QMA ion exchange column with 1.0 mL of K222-K_2_CO_3_ solution and added to the reaction flask. The eluted radioactivity was heated to 95 °C and dried under a compressed nitrogen purge as well as under negative pressure. Then, acetonitrile was added to the reaction tube several times, and ^18^F-fluoride ion was further dried by azeotropic evaporation of water–acetonitrile (95 °C under a compressed nitrogen purge and reduced pressure) for 7 min. After completion, the heating temperature was raised to 100 °C under reduced pressure to obtain the dried K222-^18^F-potassium fluoride. The precursor solution (about 2 mg dissolved in 1.5 mL dimethyl sulfoxide, Sigma-Aldrich, Saint Louis, MO, USA) was added into the reaction flask containing dry K222-^18^F-potassium fluoride, and the resulting reaction mixture was heated to 110 °C. Compressed nitrogen was slowly introduced into the reaction mixture while maintaining the heating temperature at 110 °C for at least 5 min. 3 N HCl solution (0.8 mL) was added to the reaction mixture, and the reaction mixture was heated to 120 °C for 0.5 min. After completion, the reaction mixture was cooled to 60 °C, and then 5.0 mL of a 0.5 N NaOH solution was added. Next, the resulting solution was then aspirated into a syringe and diluted with 3% sodium ascorbate. The solution in the syringe was passed through a tC18 column (Agilent Technologies, Santa Clara, CA, USA), and then the cartridge was washed with 3% w/v sodium ascorbate water for injection solution. The crude florzolotau product was eluted from the tC18 column with ethanol. The obtained florzolotau solution was loaded onto a semi-preparative high-performance liquid chromatography (HPLC, Agilent 1260, Agilent Technologies, Santa Clara, CA, USA) column for purification. The synthesis of florzolotau was done 3 times, and the yield was 43.7 ± 4.1% and the activity was 199.7 ± 0.9 MBq/mL at the end of synthesis.

### Study schedule

The study procedure and schedule are outlined in Table 1. A subject was tentatively enrolled in the study between Day-28 and Day-14 after vital signs, ECG, and laboratory test, and the expected date of PET scan was scheduled. Florzolotau (185 MBq at the calibration time) was administered as a single intravenous injection. Actual injection doses for subject 1001, 1002, and 1004 were 194.9, 195.7, and 194.4 MBq, respectively (5.27 ± 0.02 mCi). Whole-body PET scans were acquired at Session 1 to Session 6. Venous blood and urine were also collected for pharmacokinetic measurements, and those collected after image acquisition of Session 6 were also used for the laboratory test including hematological parameters, blood biochemical test, coagulation test, and urinalysis.

**Table 1:** Study schedule

	Screening	Treatment period			Follow-up
Study day	Day-28 ~ -1	Day 1			Day 2
		Pre-dose	Dosing	Post-dose	
Informed consent	X				
Interim enrollment	X				
Enrollment		X			
Eligibility check	X	X			
Medical history	X	X			
Medication history	X	X			
Body height and weight	X	X			
Physical examination	X	X			
Vital signs^a^	X	X		X	
Body temperature^b^		X		X	
ECG^a^	X	X		X	
Laboratory test^c^	X			X	
Blood collection for PK^d^				X	
Urine collection for PK^e^				X	
Florzolotau injection			X		
PET-CT				X	
Adverse events	X^f^	X^f^	X	X	X
Concomitant medications				X	

^a^Vital signs (systolic/diastolic blood pressure, heart rate, and respiratory rate) and ECG were taken at screening, prior to and immediately after administration of florzolotau and after image acquisition of Session 6

^b^Body temperature was taken prior to administration of florzolotau and after image acquisition of Session 6

^c^Including hematology, blood biochemical test, coagulation test and urinalysis. Serological tests were also performed at screening. Blood and urine were collected after image acquisition of Session 6

^d^Approximately 2 mL of venous blood was collected immediately after administration of florzolotau, after completion of each whole-body scan in Session 1 (started 15, 25, 36 and 47 min after injection) and after image acquisition of Session 2 through 6 (approximately 20 mL in total)

^e^Subjects were urged to urinate before administration of the study drug. Urine was collected after image acquisition of Session 6, and urination at other time points were also allowed (urine collection after Session 6 was mandatory)

^f^Medical events occurred after the time of informed consent and before study drug administration were recorded as medical history or comorbid diseases

### Whole-body PET imaging

Serial PET scans of the whole body (top of head to mid-thighs) were acquired PET/computed tomography (CT) scanner (Biograph Truepoint 16, Siemens Healthcare, Erlangen, Germany) to assess the bio-distribution and radiation dosimetry of florzolotau in healthy male subjects. Four whole-body PET scans for 90 s/bed position were acquired after completion of vital signs and ECG examinations immediately after the florzolotau administration, the scan was started 15–16 min after the injection (Session 1). Following an approximately 30 min break, two whole-body PET scans for 2 min/bed position were acquired (Session 2). Since then, one whole-body PET scan for 4 min/bed position was repeated at an approximately 30 min break (Session 3–6). A transmission CT scan for positioning and attenuation correction was performed before commencing the Session 1. After that, when the subject moved, a transmission CT scan was performed before or after the imaging session as needed. The acquired emission data were reconstructed using the 3-dimensional ordered subset expectation maximization method with attenuation, random, and scatter correction and without the point spread function correction. The reconstruction parameters were matrix size of 256 × 256 with 21 subsets and 2 iterations.

### Dosimetry analysis

Reconstructed data were analyzed using PMOD software (version 4.0, PMOD Technologies, Zurich, Switzerland). Three-dimensional volumes of interest (VOIs) were constructed on the PET images to include all organ activities. The VOIs of the target organs including the brain, lungs, heart wall, stomach wall, liver, gallbladder, spleen, pancreas, upper large intestine, lower large intestine, rectum, kidneys, and urinary bladder wall were placed using the HERMES Data Analysis Application Hybrid Viewer Dosimetry version 4.0 (Hermes Medical Solutions, Stockholm, Sweden). Regarding the upper large intestine and the lower large intestine, the VOIs were placed from the ileocecal region to the transverse colon and from the descending colon until the sigmoid colon, respectively. The anatomical information from the CT data and the human whole-body reference atlas was used to delineate subject specific VOIs. Average counts for the VOIs were determined at each whole-body scan using the PET emission data.

Decay-corrected activity in each organ was expressed as a percentage of the injected dose (%ID) and plotted against time to generate time–activity curves (TACs). The numbers of disintegrations, formerly referred to as residence time, were calculated from the area under the non-decay-corrected TACs from time zero to infinity with trapezoid method (or model-fitting), multiplied by the volume of the organ. The residence times were entered in OLINDA/EXM software (version 2.1, Hermes Medical Solutions, Stockholm, Sweden), and the absorbed doses in each organ and whole-body effective dose were calculated using the following formulas.

$${\text{Absorbed dose }} = {\text{ Residence time }} \times {\text{S - factor}}/{\text{Administered dose}}{.}$$

$${\text{Effective dose }} = {\Sigma }_{{\text{T}}} {\text{(Equivalent dose*}} \times {\text{Tissue weighting factor)}}$$

$$\begin{gathered} {\text{*Equivalent dose }} = {\text{ Absorbed dose}} \times {\text{Radiation weighting factor}} \hfill \\ \left( {{\text{Radiation weighting factor }} = { 1}} \right) \hfill \\ \end{gathered}$$

S-factors are implemented within the OLINDA/EXM software. The International Commission on Radiological Protection (ICRP) adult male phantom model (70 kg and 170 cm tall, ICRP-89) was used to calculate the absorbed doses, and the ICRP-103 tissue weighting factors were used to calculate the effective dose. Calculations were performed without modeling of urinary bladder voiding.

### Measurement of radioactivity in blood and urine

Before the tracer administration, another venous route was secured on the other side forearm than the injection side. A total of 10 blood samples were taken from the venous route: immediately, approximately 10, 20, 30, and 40 min after the injection, and after each following session. Radioactivity in whole blood was measured using a gamma-counter (ARC-8001, Hitachi Aloka Medical, currently Fujifilm Healthcare Manufacturing, Minamiashigara, Japan). Subjects were urged to urinate before administration of the study drug. Then, urine was collected in principle after image acquisition of Session 6, and urination after image acquisition of Sessions 1, 2, 3, 4, and 5 was also allowed when a subject felt a need to urinate. Urine was collected after Sessions 2 and 6 for subject 1001 and after Sessions 4 and 6 for subject 1002 and 1004. Urine radioactivity was measured using the gamma-counter as well.

### Safety measurements

Vital signs (systolic/diastolic blood pressure, heart rate, and respiratory rate) and ECG were taken at screening, prior to and immediately after administration of florzolotau and after image acquisition of Session 6. The laboratory tests including hematology, blood biochemical test, coagulation test, urinalysis, and serological tests were also performed at screening and after imaging acquisition of Session 6.

## Results

Three male subjects were enrolled in this study. All subjects received a single intravenous dose of florzolotau and included in the safety, pharmacokinetics, and dosimetry datasets. The statistical analyses performed in this study were specified in the statistical analysis plan finalized prior to the database lock. SAS 9.4 and SAS 9.5 were used for the statistical analysis.

### Bio-distribution and dosimetry

**Fig. 1:**
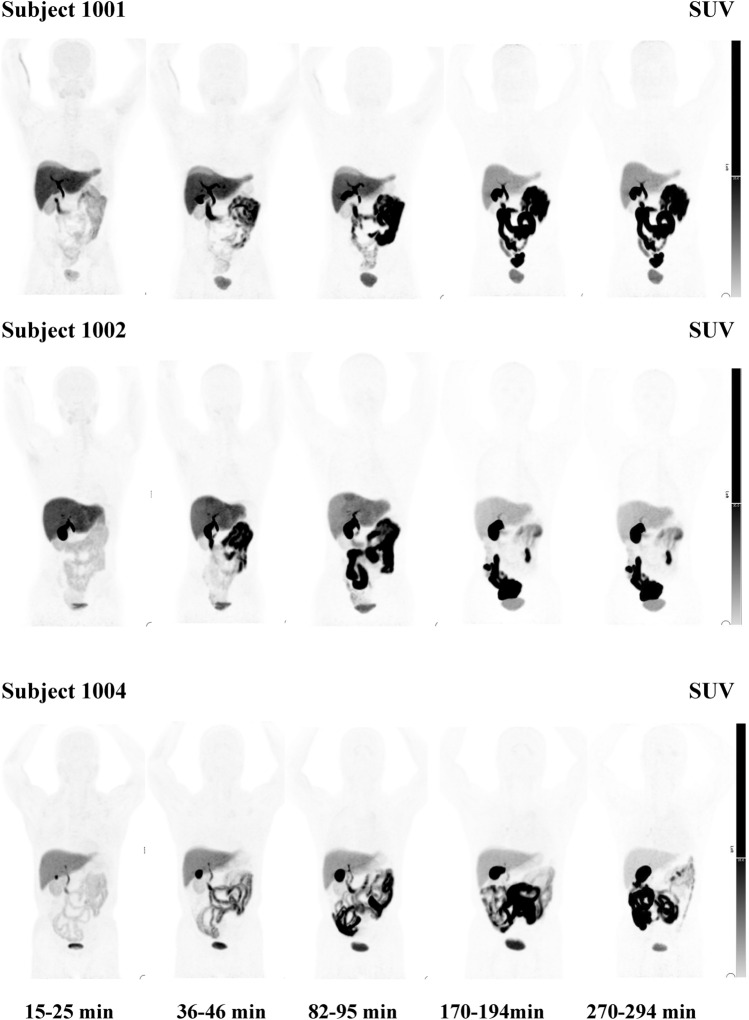
Florzolotau whole-body PET images in 3 healthy subjects. Images show distribution changes over to 4.9 h after the injection in 3 subjects. The bars on the right-side show SUV units. The center value of the SUV scales is 20

Serial whole-body PET images of individual subjects are presented in Fig. 1. Uptake of the radiotracer in the brain, lung, heart, liver, kidney, and pancreas was high at 10–25 min after the florzolotau injection, then the radioactivity gradually decreased with time. The radioactivity in gallbladder wall and intestine was high at 10–25 min, and the radioactivity increased with time, the uptake peaked 1–2 h and then gradually declined.

**Fig. 2:**
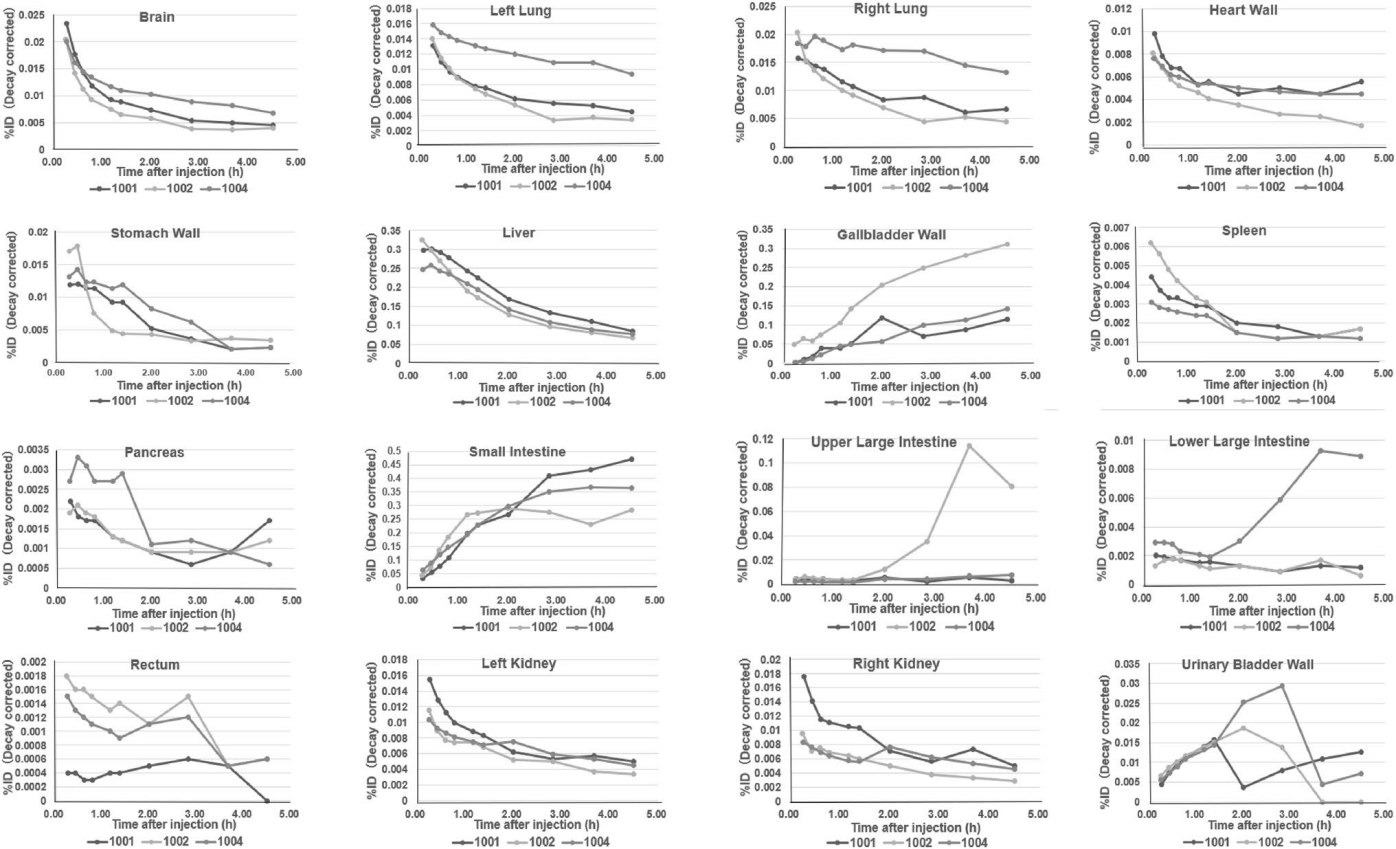
Time–activity curve (decay-corrected) after florzolotau injection. Decay-corrected activity is shown as percentage of the injected radiological activity as %ID

The time–activity curves of the decay-corrected %ID of various organs for 3 subjects are shown in Fig. 2. Organ showing the highest mean initial ^18^F activity uptake at the initial imaging time point of 0.25–0.42 h was the liver (29.0 ± 4.0%). The %ID was also high in the intestine (4.69 ± 1.65%) and the brain (2.13 ± 0.18%). The organs with a fast wash-in and washout were brain, lungs, heart, and kidneys. The radioactivity in the intestine and gallbladder was increased with time, and the radioactivity in the rectum and urinary bladder was higher at 2–3 h after the injection. The residence time for each organ is shown in Table 2. Among the mean residence times of organs, the largest average retention time was the liver of 0.47 ± 0.05 h, followed by the small intestine of 0.43 ± 0.06 h and the gallbladder wall of 0.29 ± 0.09 h. The bladder and the bowel showed substantial uptake from 15 min until the end of the study due to urine accumulation and radioactivity elimination through the gastrointestinal tract. The residence time was short in brain, lungs, heart wall, kidneys, spleen, pancreas, upper large intestine, lower large intestine, rectum, and urinary bladder wall.

**Table 2:** Residence time for each subject after injection of florzolotau in 3 healthy subjects

	Residence Time (MBq·h/MBq)				
Subject ID	1001	1002	1004	Mean	SD
Brain	0.0245	0.0193	0.0284	0.0241	0.0046
Left lung	0.0187	0.0160	0.0313	0.0220	0.0082
Right lung	0.0085	0.0281	0.0435	0.0267	0.0175
Heart wall	0.0151	0.0103	0.0138	0.0131	0.0025
Stomach wall	0.0179	0.0148	0.0224	0.0184	0.0038
Liver	0.4900	0.5133	0.4153	0.4729	0.0512
Gallbladder wall	0.3123	0.3612	0.1950	0.2895	0.0854
Spleen	0.0061	0.0030	0.0046	0.0046	0.0016
Pancreas	0.0027	0.0017	0.0049	0.0031	0.0016
Small intestine	0.4756	0.4666	0.3602	0.4341	0.0642
Upper large intestine	0.0021	0.0366	0.0067	0.0151	0.0187
Lower large intestine	0.0036	0.0022	0.0059	0.0039	0.0019
Rectum	0.0003	0.0028	0.0026	0.0019	0.0014
Left kidney	0.0201	0.0101	0.0180	0.0161	0.0053
Right kidney	0.0230	0.0084	0.0169	0.0610	0.0073
Urinary bladder wall	0.0148	0.0094	0.0182	0.0141	0.0044

**Fig. 3:**
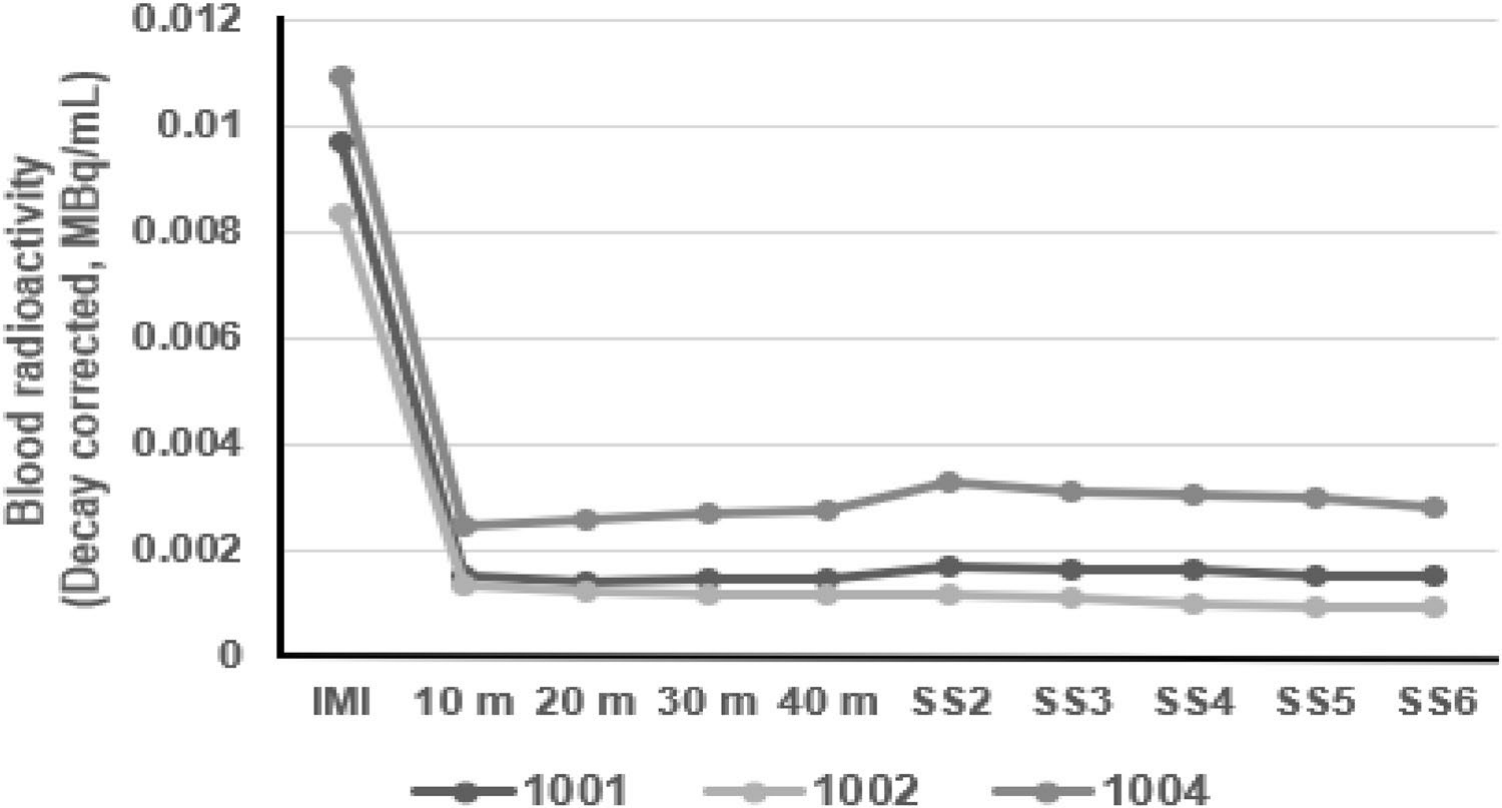
Decay-corrected radioactivity in blood. Blood collection was done after the PET scan. Session 2 (SS2), 3 (SS3), 4 (SS4), 5 (SS5) and 6 (SS6) were started at 70, 120, 170, 220 and 270 min after the injection. Blood sample was taken at 1 min after the injection for subject 1001 and 1004 and at 2 min after the injection for subject 1002. *IMI* immediately after injection, *m* minutes after injection

The decay-corrected radioactivity in blood samples of three subjects is shown in Fig. 3. The mean radioactivity taken immediately after florzolotau injection (1–2 min after the injection) was 0.0097 ± 0.0013 MBq/mL, and the level was steeply decreased at 10 min after the injection to 0.0018 ± 0.0006 MBq/mL and remained until at the end of Session 6 (0.0018 ± 0.0010 MBq/mL). The blood half-life of the compound was 16.4 ± 5.4 h. The mean total decay-correct radioactivity in urine until the end of Session 6 (for 270 min after the injection) was 7.49 ± 0.73 MBq. Excretion of ^18^F activity by renal pathway was 3.84% of ID.

Absorbed doses in each organ and whole-body effective doses were calculated using OLINDA/EXM (version 2.0). The results are summarized in Table 3. The highest absorbed dose was 508 μGy/MBq of the gallbladder wall, followed by the liver of 79.4 μGy/MBq, pancreas of 42.5 μGy/MBq, upper large intestine of 34.2 μGy/MBq, and lower large intestine of 24.1 μGy/MBq. The effective dose was calculated as 19.7 μSv/MBq according to tissue weighting factor reported by ICRP-103. Based on the results of this experiment, the subject will receive and effective dose of approximately 3.61 mSv when 185 MBq florzolotau was given to a male subject.

**Table 3:** Absorbed doses in various organs after injection of florzolotau in 3 healthy subjects

	Absorbed Dose (μGy/MBq)				
Subject ID	1001	1002	1004	Mean	SD
Brain	5.1	4.0	5.8	4.9	0.9
Lung	11.2	10.5	17.6	13.1	3.2
Heart wall	11.4	10.5	15.5	12.5	2.7
Stomach wall	19.3	18.1	21.5	19.6	1.7
Liver	81.5	93.3	63.3	79.4	15.1
Gallbladder wall	546	629	348	508	144
Spleen	9.8	6.3	13.5	9.9	3.6
Pancreas	41.1	49.6	36.7	42.5	6.6
Small intestine	127	119	87	111	21
Upper large intestine	27.4	49.7	25.5	34.2	13.5
Lower large intestine	24.5	22.9	24.9	24.1	1.1
Rectum	15.4	10.0	10.3	11.9	3.0
Kidney	9.1	5.1	7.1	7.1	3.0
Urinary bladder wall	14.2	11.5	17.0	14.2	2.8

ICRP-89 adult male phantom model

### Safety

There have been no deaths or discontinuations due to adverse events. No serious adverse event was noted in this study. Evaluation of vital signs and ECG revealed no statistically significant findings nor result in clinically meaningful individual changes.

Vital signs (systolic/diastolic blood pressure, heart rate, and respiratory rate) and ECG were examined at baseline (screening period), prior to and immediately after administration of the study drug and after the final PET imaging session on Day 1. There were no clinically significant changes in any vital signs (body temperature, Systolic/diastolic blood pressure, heart rate, and respiratory rate) and ECG in any subjects after study drug administration.

Blood for clinical laboratory tests was collected at baseline (screening period) and after the final PET imaging session on Day 1. Only subject No. 1004 experienced 6 adverse events. All the adverse events were mild, which were urine protein, increases in aspartate aminotransferase, alanine aminotransferase, gamma-glutamyl-transferase, dyslipidemia, and increase in blood bilirubin, which were recovered at the biochemical test conducted 19 days after the injection. These adverse events were judged as not related to the study drug.

## Discussion

The safety, bio-distribution, and internal radiation dosimetry profiles in Japanese healthy subjects following the injection of florzolotau have been shown. Florzolotau intravenous injection was well tolerated in healthy male Japanese subjects as well. Although the subject 1004 showed transient increase in hepatic enzymes and blood bilirubin and dyslipidemia, it was suspected to be related to excessive alcohol intake during New Year holidays between the screening and test days.

The uptake pattern in the organs was similar to those of the former report using black and Asian healthy subjects [19, 20]. In the former Asian study, there was no significant difference in the effective dose and organ distribution between male and female subjects (unpublished data). Brain uptake of florzolotau was high at early stage after administration of florzolotau reflecting high blood concentration, and the radioactivity was quickly eliminated from the brain, which seems to be suitable pharmacokinetic profile as a tau PET ligand. Rapid clearance from circulation was observed with approximately 10% of the administered dose remaining at 1–2 min after the injection, and less than 2% at 10 min or more longer time periods. The rapid clearance from circulation is considered due to its high tissue penetration, and the clearance may be faster than β-amyloid tracers of florbetapir and flutemetamol [21, 22]. The total cumulative radioactivity present in urine was low at 3.84%ID, comparable to 4.0–6.0% in the US study (unpublished data).

The uptake of florzolotau at 15 min after injection was high in the liver, intestine, brain, kidney, and lungs, especially the uptake in the liver was highest (29.0%ID) followed by the intestine (4.69%ID). The elimination of florzolotau was exhibited mainly via hepatobiliary pathway. The highest absorbed dose was observed in the gallbladder wall followed by the liver, pancreas, and intestines. Similar elimination pattern with the highest absorbed dose in the gallbladder wall was observed in other amyloid and tau PET tracers including florbetapir, flutemetamol and THK-5351 [21–23], and tau PET tracer MK-6240 [24, 25]. The effective dose of florzolotau was calculated as 19.7 μSv/MBq according to tissue weighting factor reported by ICRP-103. The effective dose was 3.61 mSv when 185 MBq florzolotau was given to a subject. The mean effective dose of florzolotau in black subjects was 34.6 μSv/MBq which was higher than that in Japanese subjects. The effective dose of florzolotau was lower than those of β-amyloid tracers of florbetapir (5.5 mSv/370 MBq) [21] and flutemetamol (6.5 mSv/185 MBq) [22] in healthy Japanese subjects, and tau tracers of THK-5351 (8.4 mSv/370 MBq) [23], flortaucipir (4.7 mSv/185 MBq) in healthy Asian subjects [26], MK-6240 (5.0 mSv/185 MBq) in healthy elderly Japanese subjects [25], and PI-2620 (6.1 mSv/185 MBq) in healthy male subjects [27]. Also, the effective dose of florzolotau was well below the whole-body single dose limit of 30 mSv specified by the FDA [28]. It was concluded florzolotau (185 MBq) was well tolerated and no particular concern about exposure dose of nuclear medicine examinations.

## Conclusion

Florzolotau injection was well tolerated in healthy Japanese subjects. The effective dose was 19.7 μSv/MBq, and 3.61 mSv when 185 MBq florzolotau was given to a healthy male Japanese subject.

## Data Availability

The datasets generated and /or analyzed during the current study are available from the corresponding author on reasonable request.
