# Correction: Radiation dosimetry and pharmacokinetics of the tau PET tracer florzolotau (18F) in healthy Japanese subjects

**DOI:** 10.1007/s12149-024-01917-5

**Published:** 2024-03-07

**Authors:** Masaomi Miyamoto, Chio Okuyama, Shinya Kagawa, Kuninori Kusano, Masaaki Takahashi, Keisuke Takahata, Ming-Kuei Jang, Hiroshi Yamauchi

**Affiliations:** 1 APRINOIA Therapeutics Inc., Tokyo, Japan; 2 Shiga Medical Center Research Institute, Moriyama, Japan; 3 Institute for Quantum Medical Science, Quantum Life and Medical Science Directorate, National Institutes for Quantum Science and Technology, Chiba, Japan; 4 Department of Psychiatry, Graduate School of Medicine, Kyoto University, Kyoto, Japan

**Correction: Annals of Nuclear Medicine (2023) 37:300–309** 10.1007/s12149-023-01828-x

In the sentence beginning ‘The effective dose was…’ in this article [in “Abstract”, under the heading ‘Conclusion’], the value ‘3.61 mSv’ should have read ‘3.64 mSv’.

In the sentence beginning ‘Based on the results of this experiment, …’ in this article [in page 305], the value ‘3.61 mSv’ should have read ‘3.64 mSv’.

In Table 2 of this article, the data in the ‘Right kidney’ headed ‘Mean’, the value ‘0.0610’ should have read ‘0.0160’.

In the sentence beginning ‘The effective dose was…’ in this article [in page 307], the value ‘3.61 mSv’ should have read ‘3.64 mSv’.

In the sentence beginning ‘The effective dose was 19.7 μSv/MBq, …’ in this article [in page 308], the value ‘3.61 mSv’ should have read ‘3.64 mSv’.

